# The Microbial Trojan Horse and Antimicrobial Resistance: Acanthamoeba as an Environmental Reservoir for Multidrug Resistant Bacteria

**DOI:** 10.1111/1462-2920.70193

**Published:** 2025-10-29

**Authors:** Ronnie Mooney, Erin Corbett, Elisa Giammarini, Kiri Rodgers, Carla Donet, Ernest Mui, Arhama T. A. Ansari, Ayush Ransingh, Pradnya S. Vernekar, Harleen K. Walia, Jyoti Sharma, John Connolly, Andrew Hursthouse, Suparna Mukherji, Soumyo Mukherji, Fiona L. Henriquez

**Affiliations:** ^1^ Department of Civil and Environmental Engineering University of Strathclyde Glasgow UK; ^2^ Joint Nature Conservation Committee (JNCC) Inverdee House Aberdeen UK; ^3^ School of Health and Life Sciences University of the West of Scotland Paisley UK; ^4^ Environmental Science and Engineering Indian Institute of Technology Bombay Mumbai India; ^5^ Glasgow School for Business and Society Glasgow Caledonian University Glasgow UK; ^6^ School of Computing, Engineering & Physical Sciences University of the West of Scotland Paisley UK; ^7^ Department of Biosciences and Bioengineering Indian Institute of Technology Bombay Mumbai India

**Keywords:** *Acanthamoeba*, antimicrobial resistance, intracellular, microbiome, potentially toxic elements, Symbiosis

## Abstract

Antimicrobial resistance (AMR) is shaped by environmental pressures, yet the role of microbial predators such as *Acanthamoeba* in resistance dynamics remains poorly characterized. In this study, *Acanthamoeba*‐associated bacterial communities (AAB) exhibited significantly higher multidrug resistance than sediment‐associated bacterial communities (SAB) in a polluted estuarine system. All isolated amoebae belonged to the T4 genotype, suggesting selection for resilient host organisms. AAB displayed elevated multiple antibiotic resistance (MAR) indices and increased resistance to multiple antibiotic classes, particularly aminoglycosides, macrolides, fluoroquinolones and β‐lactams. Correlation analysis revealed that resistance in AAB, but not SAB, was associated with potentially toxic elements (PTEs) known to influence phagocyte survival, including arsenic, vanadium, and calcium. These elements may select for traits that confer metal and antibiotic resistance. The findings support a model where protists act as selective environments for AMR, favoring bacteria that possess enhanced tolerance mechanisms. This work provides the first direct evidence linking PTE exposure to the intracellular resistome of *Acanthamoeba*‐associated bacteria. It underscores the need for AMR monitoring frameworks that include protist‐bacteria interactions, with implications for One Health and environmental risk assessment strategies. Moreover, this approach is scalable for application in low/middle‐income countries, where AMR burden is greatest and surveillance capacity remains limited.

## Graphical abstract

Bacteria associated with *Acanthamoeba* exhibit greater antibiotic resistance than those from sediment and, on average, show a higher likelihood of multi‐antibiotic tolerance, reflected in significantly elevated multiple‐antibiotic resistance (MAR) index values. Potentially toxic elements driving resistance also differ depending on the type of survival; *Acanthamoeba*‐associated or sediment associated.

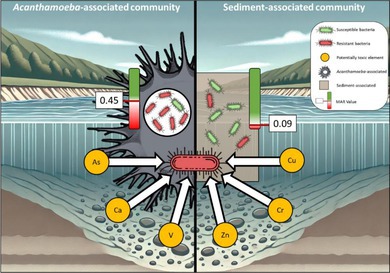

## 1 Introduction

Antimicrobial resistance (AMR) is one of the most pressing global health challenges of our time. The ability of microorganisms to resist previously effective therapeutics compromises the treatment of infectious diseases, leading to prolonged illnesses, higher medical costs and increased mortality. Bacterial AMR alone was directly responsible for the death of 1.27 million people globally in 2019 and contributed to approximately 5 million deaths (Murray et al. 2022). Environmental reservoirs are increasingly recognised as key contributors to the persistence and spread of AMR, particularly in polluted systems (Rodgers et al. 2019; Hubeny et al. 2021; Abd‐El‐Aziz et al. 2017; Acar Kirit et al. 2022; Goh et al. 2024).

Anthropogenic activities are major drivers of AMR in the environment, with pollutants including antibiotics, potentially toxic elements (PTEs), and industrial chemicals creating selective pressures that favor the survival and proliferation of resistant microorganisms (Abd‐El‐Aziz et al. 2017; Ashbolt et al. 2013; Li et al. 2024; Kusi et al. 2022; Knapp et al. 2017). Within these complex ecosystems, interactions between microorganisms such as bacteria and protists can potentially influence resistance dynamics. Free‐living amoebae (FLA), particularly those in the genus *Acanthamoeba*, are widely distributed in terrestrial and aquatic habitats and play dual roles as both predators and hosts of bacteria (Choi et al. 2024; Henriquez et al. 2021; Mooney et al. 2024; Clarholm 1981). These amoebae can survive under extreme environmental conditions, including desiccation, ultraviolet radiation, and exposure to biocides and antibiotics (Sriram et al. 2008; Johnston et al. 2009; Coulon et al. 2010; Lorenzo‐Morales et al. 2015). The predatory behavior of *Acanthamoeba* involves the engulfment of bacteria through phagocytosis (Chambers and Thompson 1976). While this process typically results in bacterial degradation, certain bacteria, known as amoeba‐resistant bacteria, have evolved mechanisms to evade digestion and survive within the amoebae, using them as protective reservoirs (Mooney et al. 2024; Rowbotham 1980; Schmitz‐Esser et al. 2008). The association between *Acanthamoeba* and amoeba‐resistant bacteria has significant implications for the spread of AMR (Henriquez et al. 2021; Vingataramin et al. 2025). Intracellular bacteria are shielded from environmental stressors such as antibiotics, PTEs and disinfectants, enabling their persistence and proliferation, and studies have demonstrated that bacteria within *Acanthamoeba* can exhibit enhanced resistance traits, complicating efforts to control AMR in environmental settings (Giammarini et al. 2025). This study focuses on the ecologically significant Vashi Creek, Mumbai (Corbett et al. 2025), and the potential role of anthropogenic pollutants and intracellular survival on antimicrobial resistance.

The Vashi Creek, an estuarine waterway in Mumbai, India, is part of the larger Thane Creek system and serves as a vital ecological and economic zone (Corbett et al. 2025; Aa et al. 2022; Chaudhari‐Pachpande and Pejaver 2016; Singh et al. 2023). However, it is also subject to significant anthropogenic inputs, including untreated sewage, industrial discharges, and agricultural runoff. These inputs introduce AMR genes to the environment (Ju et al. 2019) as well as high levels of pollutants, including antibiotics, PTEs and organic waste, creating conditions conducive to the proliferation of AMR (Rodgers et al. 2019; Hubeny et al. 2021; Abd‐El‐Aziz et al. 2017; Ashbolt et al. 2013; Li et al. 2024; Kusi et al. 2022; Knapp et al. 2017; Laxminarayan et al. 2013; Shallcross and Davies 2014; Tamma et al. 2017). The dynamic nature of the estuarine environment, characterized by fluctuating salinity, nutrient levels and pollutant concentrations, further influences microbial community structure and resistance dynamics (Kaviani Rad et al. 2022; Windels et al. 2024). The relevance of Vashi Creek as a study site for AMR is underscored by its role as a repository for pollutants from Mumbai, one of India's most densely populated urban centers. With limited wastewater treatment infrastructure, large volumes of domestic and industrial effluents are discharged directly into the creek. These effluents introduce a diverse array of pollutants that exert selective pressure on microbial communities, which in turn can enrich resistance genes, reflecting the influence of anthropogenic stressors (Hubeny et al. 2021; Corbett et al. 2025).

In addition to its role as a hotspot for AMR, Vashi Creek provides a unique context for studying *Acanthamoeba*‐bacteria interactions. The creek's microbial ecosystem is shaped by a combination of natural estuarine processes and anthropogenic impacts, creating opportunities for complex microbial interactions. *Acanthamoeba*, with their ability to harbor intracellular bacteria and survive in extreme environments, play a critical role in this dynamic (Mooney et al. 2024; Rayamajhee et al. 2024, 2021). These amoebae not only facilitate the persistence of amoebae‐resistant bacteria but also act as vectors for their dissemination. The potential for *Acanthamoeba* to transport resistant bacteria across different environmental and clinical spheres highlights their importance in the ecology of AMR, reinforcing the need for a One Health approach to address the interconnected risk to human, animal, and environmental health. This is particularly pertinent in low‐ and middle‐income countries (LMICs), where environmental AMR pressures are intensified by inadequate wastewater treatment, industrial pollution, and high population density. Moreover, resource constraints often preclude genomic surveillance, highlighting the need for robust, low‐cost phenotypic approaches to AMR risk profiling. Intracellular bacteria harbored within amoebae are less detectable using conventional microbiological methods, posing challenges for monitoring and mitigation efforts (Henriquez et al. 2021; Mooney et al. 2024, 2025). Additionally, the potential to release viable bacteria into the environment during trophozoite reactivation raises concerns about their role in the dissemination of AMR across different ecological and geographical scales.

This study investigates the role of intracellular survival within *Acanthamoeba* in shaping environmental AMR under pollution stress. By characterising the resistance profiles between *Acanthamoeba*‐associated bacterial (AAB) communities and sediment‐associated bacterial (SAB) communities, we seek to determine the risk from intracellular survival on AMR. Specifically, this research will focus on assessing the resistance profiles of bacteria associated with *Acanthamoeba*. Our findings will provide critical insights into the environmental reservoirs of AMR and inform strategies for mitigating its impact. Briefly, we demonstrate that *Acanthamoeba* can tolerate sediments high in several potentially toxic elements (e.g., Arsenic, Chromium, Nickel), and the bacterial communities associated with them possess much higher tolerances to antibiotics. Despite extensive research on AMR in environmental bacteria, no study has systematically examined how PTEs shape intracellular bacterial resistomes. This study provides the first direct evidence of this association, revealing novel selective pressures that may drive AMR evolution. By addressing these interactions in the context of a polluted estuarine ecosystem, this study aims to contribute to the broader understanding of AMR dynamics and their implications for public and environmental health.

## 2 Experimental Procedures

The study was intentionally designed to use culturable bacterial isolation and phenotypic resistance profiling, which offer an accessible and feasible alternative to metagenomic or high‐throughput approaches. This decision reflects the need for scalable, resource‐accessible methodologies suitable for use in LMICs, where environmental AMR is often under‐surveilled despite bearing a disproportionate disease burden. Our aim is to produce data and tools that are applicable in real‐world, low‐resource settings.

### 2.1 Sampling

**FIGURE 1:**
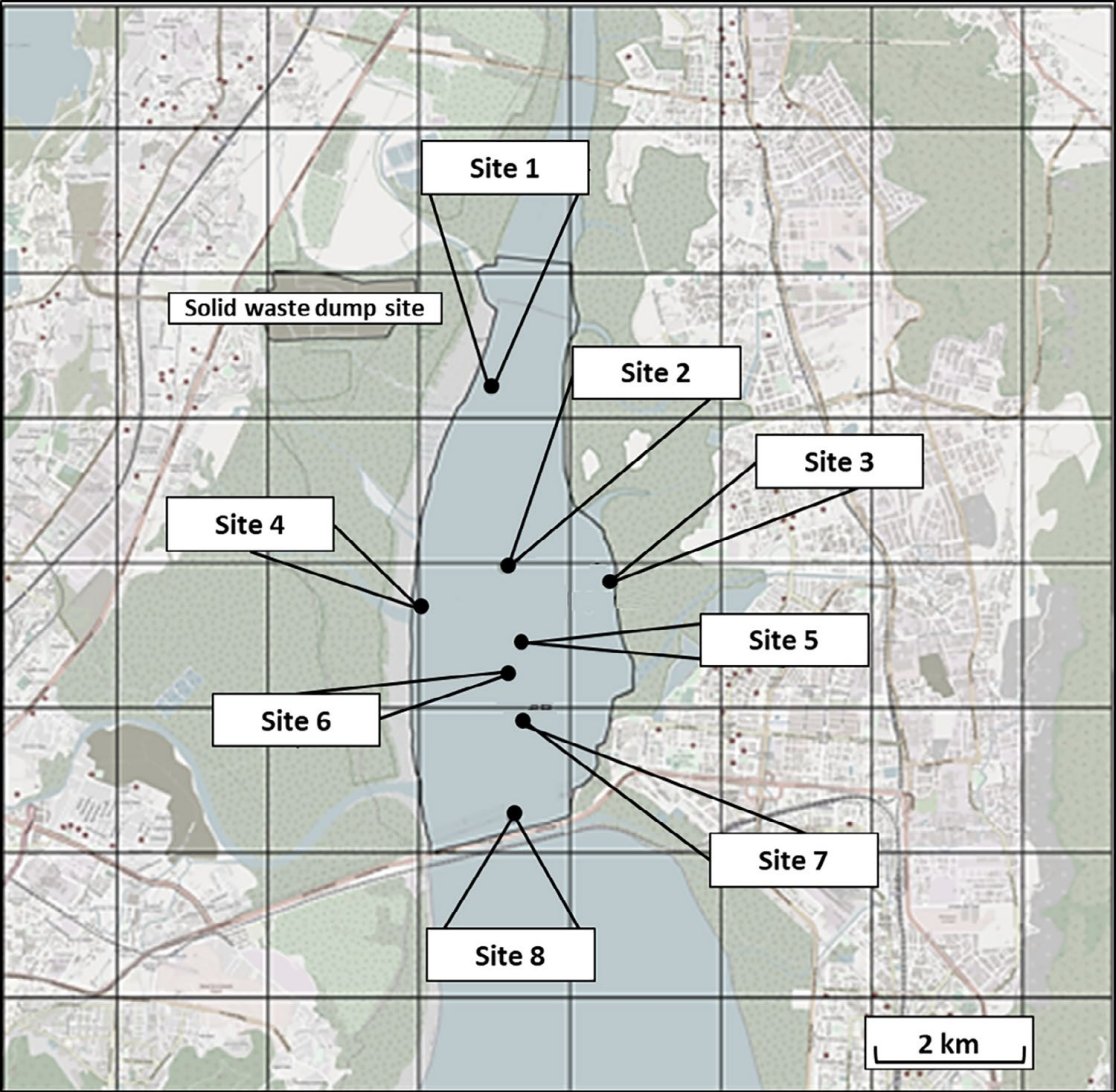
Vashi Creek with sample sites used in this study highlighted (Sites 1–8).

Sediment samples were collected from the Vashi Creek, Mumbai, India during three sample events; sample event 1 (SE1) was undertaken in November 2021; sample event 2 (SE2) was undertaken in April 2022; and sample event 3 (SE3) was undertaken in April 2023. Vashi Creek is an estuarine waterway located where the Thane Creek meets the Arabian Sea and is subject to significant tidal influence. A total of eight locations within the creek were chosen for sampling; Figure 1; Site 1 (19°5′30.3, 72°59′1.68″); Site 2 (19°5′19.5, 72°57′42.00″); Site 3 (19°5′3.98, 72°58′25.00″); Site 4 (19°5′36.78, 72°58′17.08″); Site 5 (19°6′51.98, 72°58′11.50″); Site 6 (19°4′52.00, 72°58′17.98″); Site 7 (19°4′31.01, 72°58′25.00″); Site 8 (19°3′51.00, 72°58′21.00″). Sediment was collected using a Van Veen grab sampler, and samples were stored at 4°C prior to analysis and processed within a reasonable time frame, although we acknowledge that refrigeration may reduce bacterial diversity and alter community structure.

### 2.2 Geochemical Analysis

Replicate aliquots (*n* = 3) of 0.4 ± 0.05 g of air‐dried sample were prepared for elemental analysis by microwave‐assisted digestion (Ethos Easy) with 2 mL hydrogen peroxide and 5 mL nitric acid (10‐min ramp to 160°C, 10‐min ramp to 200°C, 15‐min hold at 200°C, 1800 W). The Certified Reference Material LGC6187 River Sediment (LGC Standards) was included in the digestion method. Digests were made up to 50 mL using UHP water and filtered in situ using 0.45 μm PTFE filters (FilterMate, Environmental Express, USA). Elements were determined by Inductively Coupled Plasma—Atomic Emission Spectrometry and Mass Spectrometry (Thermo X‐Series II) as described previously (Rodgers et al. 2020). A calibration series of multi‐element standard and reference material (BCR320R‐40G—channel sediment) was determined regularly (every 60 samples + linearity *r* ^2^ > 0.99) with indium used as the internal standard. Each determination was made in quadruplicate and dilutions were made if necessary. Isotopes used for measurement were ^75^As, ^59^Co, ^52^Cr, ^63/65^Cu, ^60^Ni, ^206/207/208^Pb and ^66/67^Zn. The pH was measured using an Orion VersaStar Pro meter (Thermo Scientific).

### 2.3 Isolation and Sequencing of *Acanthamoeba* Species

*Acanthamoeba* were isolated as reported previously by streaking 50–100 mg of sediment onto non‐nutrient agar with Page's saline, allowing a minimum of 24 h for migration, identifying cysts by morphology under an inverted microscope, and transferring cells twice to fresh plates to ensure purity (Mooney et al. 2024, 2025; Page 1978). Cells were genotyped by initially extracting DNA using the Qiagen blood and tissue kit, before validating that isolates were indeed *Acanthamoeba* by amplifying the ASA.S1 region of the 18S rRNA gene specific to the genus by PCR (Schroeder et al. 2001). PCR was performed using the Expand hi‐fidelity PCR (Sigma‐Aldrich) kit as per the manufacturer's instructions, and Sanger sequencing of the amplicon was carried out by Eurofins Genomics. Data were screened using BLAST (NCBI) to verify and genotype the isolated amoebae. The evolutionary history was inferred by using the Maximum Likelihood method and Tamura‐Nei model (Tamura and Nei 1993) and the tree with the highest log likelihood was selected (Felsenstein 1985). Evolutionary analyses were conducted in MEGA11 (Tamura et al. 2021).

### 2.4 Isolation and Culture of bacteria From Sediment and amoeba Isolates

To capture different bacterial subpopulations, isolates were recovered on both nutrient‐rich LB agar (broadly permissive) and selective cetrimide agar (enriching for *Pseudomonas* spp.). Plates were incubated aerobically at 25°C for 24 h, after which colonies were sub‐cultured to ensure purity. Antibiotic panels were tailored to Gram‐staining profiles to ensure biological relevance. We recognize that the use of selective media such as cetrimide could bias the community towards more resistant taxa; therefore, resistance rates and MAR indices were analysed separately for LB‐ and cetrimide‐derived isolates. Upon successful amplification of the ASA.S1 region by PCR, the culturable intracellular bacteria from the *Acanthamoeba* isolates were recovered by physical lysis of the amoebae and subsequent growth as is detailed in section 2.5. *Acanthamoeba* were lysed by resuspending the cells in PBS and repeatedly passing the suspension through a 27‐gauge syringe until all cells were adequately lysed.

### 2.5 Antimicrobial Susceptibility Test (AST)

The AST was conducted on agar plates according to the Kirby‐Bauer Disk Diffusion Susceptibility Test Protocol as described in Hudziki (Hudzicki 2009). The test guidelines adhered to the standards set by EUCAST and CLSI (EUCAST 2023; CLSI 2021) Antibiotic susceptibility was interpreted using EUCAST breakpoint tables (version 14.0, 2024). For isolates not identified to genus level, we applied the non‐species related PK/PD breakpoints. For isolates recovered on cetrimide agar (enriching for *Pseudomonas* spp.), we applied the EUCAST *Pseudomonas* spp. breakpoints. The antibiotics tested included erythromycin (ERY—30 μg), ampicillin (AMP—10 μg), ciprofloxacin (CIP—10 μg), streptomycin (STR—25 μg), azithromycin (AZM—15 μg), sulfamethazine (SUL—30 μg), linezolid (LIN—10 μg), oxacillin (OXA—1 μg), sulfamethoxazole/trimethoprim (SXT—25 μg), enrofloxacin (ENR—5 μg), chloramphenicol (CHL—30 μg), and tetracycline (TET—30 μg). Antibiotic concentrations were selected based on the standard guidelines outlined by EUCAST and CLSI.

### 2.6 Multiple Antibiotic Resistance (MAR) Index

To determine the variability in multidrug resistance for all conditions, each isolate was scored using the Multiple Antibiotic Resistance (MAR) index (Krumperman 1983) and the range and average MAR index value were calculated. The MAR index is calculated as follows:

$$\mathrm{MAR}=\frac{a}{b}$$

where a is the number of antibiotics to which an isolate is resistant and b is the total number of antibiotics tested. An average MAR index greater than 0.2 indicates that the environment contains a high degree of multidrug resistant bacteria.

### 2.7 Assessment of Anthropogenic Pollution in Sediments

These indices were selected to provide a comprehensive assessment of sediment contamination and its potential ecological and toxicological impact. Each index offers a distinct perspective and using a combination of four mitigates biases associated with any single approach (Machado da Silva Acioly et al. 2024; Carrillo et al. 2022).

#### 2.7.1 Pollution Load Index (PLI)

The Pollution Load Index (PLI) was calculated to assess the overall contamination level in sediment samples relative to natural background levels (Tomlinson et al. 1980). The PLI is expressed as the geometric mean of the contamination factors (CF) for all analysed PTEs using the equation:

$$\mathrm{PLI}=\sqrt[{n}]{\left(\mathrm{CF}1\times \mathrm{CF}2\times \ldots \mathrm{CFi}\right)}$$

where n represents the number of PTEs studied and where CFi is the contamination factor for PTE i, defined as the ratio of the measured concentration of the PTE in sediment to its natural background concentration. A PLI < 1 indicates low contamination, 1–2 moderate contamination, 2–4 considerable contamination, and > 4 severe contamination.

#### 2.7.2 Weighted Sediment Pollution Index (SPI)

The weighted Sediment Pollution Index (SPI) evaluates sediment quality by considering both the concentrations of PTEs and their relative environmental significance (Rubio et al. 2000). It is calculated using the formula:

$$\mathrm{SPI}=\frac{\sum \left(\frac{{\text{concentration}}_{i}}{{\mathrm{TEL}}_{i}}\right)\times {\text{weight}}_{i}}{\sum {\text{weight}}_{i}}$$

where ${\mathrm{TEL}}_{i}$ is the threshold effects level (TEL) for PTE i, and ${\text{weight}}_{i}$ is the ecological importance assigned to PTE i. Weights are determined based on the toxicity and environmental risk of each PTE. The weights assigned to each PTE were 10, 20, 5, 2, 5 and 1 for As, Cd, Cr, Cu, Ni, Pb and Zn respectively, with higher weights indicating higher toxicity (Cao et al. 2023). Scoring thresholds were tailored to reflect the observed PTE concentrations within the Thane Creek and Vashi Creek and their historical distribution reported previously (Fernandes et al. 2017). Values < 1 indicate low contamination, 1–2 indicate moderate contamination, 2–3 indicate high contamination, and > 3 indicate very high contamination.

#### 2.7.3 Ecological Risk Potential (ERp) and Integrated Risk Index (RI)

The Ecological Risk Potential (ERp) quantifies the risk posed by individual PTEs in sediments (Hakanson 1980) and can be calculated using the following:

ERp=CF×Tr

where CF is the contamination factor for the PTE, and Tr is its toxic response factor, a value that reflects the PTE's toxicity and environmental sensitivity. The Integrated Risk Index (RI) is derived by summing ERp values across all analysed PTEs as follows:

$$\mathrm{RI}=\sum {\mathrm{ERp}}_{i}$$

RI provides a comprehensive measure of the cumulative ecological risk posed by all PTEs in the sediment sample. Higher RI values indicate greater ecological risk. An RI value < 150 indicates the sediment is unlikely to cause significant ecological harm, 150–300 indicates moderate ecological risk, 300–600 indicates high ecological risk, and > 600 indicates very high ecological risk (Machado da Silva Acioly et al. 2024; Dash et al. 2021).

#### 2.7.4 Normalised Toxicity Index (TI)

The Normalised Toxicity Index (TI) quantifies the potential toxicity of PTEs in sediment based on their concentrations relative to their threshold effect level (TEL) and probable effect level (PEL) (Canadian Council of Ministers of the Environment 2002; Macdonald et al. 1996). It uses well‐documented TEL and PEL values to evaluate the risk of biological effects occurring within the system and provides a consistent scale for monitoring toxicity across different PTEs and locations. It is calculated as:

$$\text{Normalised Value}=\frac{{\text{concentration}}_{i}-{\mathrm{TEL}}_{i}}{{\mathrm{PEL}}_{i}-{\mathrm{TEL}}_{i}}$$

For each PTE, concentrations below TEL are assigned a value of 0, while concentrations above PEL are capped at 1. The TI is the sum of normalised values across all PTEs in the sample:

$$\mathrm{TI}=\sum {\text{Normalised Value}}_{i}$$

Values below 1 suggest the risk of biological effects occurring is low, values 1–3 indicate moderate risk, 3–6 indicate high risk and > 6 indicate very high risk.

### 2.8 Statistical Analysis

All statistical analyses were conducted in Python (version 3.9) using the scipy, statsmodels, pandas, numpy, networkx, seaborn, and matplotlib libraries. Prior to analysis, data were screened for completeness and descriptive assumptions using Shapiro–Wilk tests and Q–Q plots for normality, and Levene's test for homogeneity of variances where applicable. For binary resistance data, group comparisons between AAB and SAB were performed using Fisher's exact test, appropriate for 2 × 2 contingency tables and small or unbalanced sample sizes. Where applicable, binomial confidence intervals were calculated using the Wilson score interval, which provides accurate bounds even for low or extreme proportions.

For continuous metrics such as the MAR index, comparisons between groups were made using the Mann–Whitney *U* test. Correlation analysis between resistance prevalence and potentially toxic element (PTE) concentrations was conducted using Pearson's correlation coefficient (r) within AAB and SAB groups separately. Correlation matrices were generated between PTE concentrations and MAR indices. Correlation coefficients were transformed using Fisher's z and weighted to account for sample size. Associations were considered exploratory: only correlations with *p* < 0.1 and |*r*| > 0.3 were retained as suggestive signals for further discussion, recognizing the limitations of multiple testing in environmental datasets. To control the false discovery rate, *p*‐values were adjusted using the Benjamini–Hochberg procedure. Correlation strength was interpreted as weak (0.3–0.399), moderate (0.4–0.599), or strong (≥ 0.6).

Network analysis was performed to explore inter‐variable relationships, using correlation matrices to construct undirected network graphs, with node centrality metrics (degree, betweenness, eigenvector centrality) computed. Visualization was achieved through force‐directed layouts in networkx. Hierarchical clustering heatmaps were generated using Ward's linkage and Euclidean/correlation‐based distance measures to identify patterns. All visualizations were generated using matplotlib and seaborn, while data pre‐processing was performed using pandas and numpy.

## 3 Results

### 3.1 Potentially Toxic Elements Are Consistently High in the Sediment of the Vashi Creek

Sediment was taken from eight sample sites in the Vashi Creek over the course of three sample events (SE 1–3), and potentially toxic elements were quantified. PTEs were abundant at all locations with some variation between sample events. Most significant differences were observed between sample events 1 and 2, with sample event 3 (Table 1). Our findings note that on average, levels of arsenic, cadmium, copper and zinc were above threshold effect levels (TEL), while levels of chromium and nickel generally exceeded the probable effect level (PEL) within Vashi Creek. Levels of lead were below the recommended TEL at almost all sites across the three sampling events, and the remaining PTEs without established TEL or PEL values were not above expected baseline levels (Corbett et al. 2025). The toxicity of the system was also assessed using PLI, weighted SPI, RI, and TI. In all calculations, SE1 and SE2 were categorised as being highly toxic. Toxicity dropped during SE3 across all metrics, with PLI, SPI, and TI suggesting the system was moderately toxic. Overall, all approaches found the system to be moderately to highly toxic, suggesting the microbial community, and ultimately the entire ecosystem, is under significant stress.

**TABLE 1:** PTE quantities measured at all sites during sample events 1–3 and toxic risk estimations.

		TEL	PEL	SE1	SE2	SE3	Average
pH				8.25 ± 0.1	8.10 ± 0.2	8.62 ± 0.1	8.33 ± 0.3
PTE (mg/kg)	Al			51,563.60 ± 2151.40	50,593.90 ± 1152.95	52,932.19 ± 2339.78	51,696.56 ± 1174.79
	As	5.9	17	8.36 ± 1.41*	7.53 ± 0.67*	5.72 ± 0.52	7.2 ± 1.35*
	Ca			25,374.66 ± 3253.99	20,476.06 ± 1703.62	22,651.99 ± 2841.40	22,834.23 ± 2454.37
	Cd	0.596	3.53	0.83 ± 0.18*	1.21 ± 0.56*	1.02 ± 0.66*	1.02 ± 0.18*
	Co			10.49 ± 1.65	9.20 ± 0.38	6.92 ± 0.25	8.86 ± 1.8
	Cr	37.3	90	152.45 ± 18.59**	157.68 ± 12.22**	117.73 ± 18.81**	142.62 ± 21.71**
	Cu	35.7	197	121.08 ± 18.39*	123.78 ± 11.91*	89.16 ± 19.47*	111.34 ± 19.25*
	Fe			61,991.23 ± 2231.92	60,214.73 ± 1277.42	62,781.10 ± 2444.01	61,662.35 ± 1314.41
	Mg			18,676.88 ± 836.04	19,055.20 ± 633.21	20,079.15 ± 763.42	19,270.4 ± 725.48
	Mn			850.64 ± 61.28	805.73 ± 68.54	697.88 ± 87.68	784.75 ± 78.51
	Ni	18	35.9	60.63 ± 18.08**	65.15 ± 2.81**	21.29 ± 1.26*	49.02 ± 24.12**
	Pb	35	91.3	19.96 ± 8.87	33.95 ± 5.96	2.47 ± 0.24	18.79 ± 15.77
	V			122.99 ± 18.27	126.93 ± 3.46	84.94 ± 4.23	111.62 ± 23.19
	Zn	123	315	174.72 ± 29.96*	195.51 ± 52.89*	139.57 ± 85.94*	169.93 ± 28.27*
Toxicity indicator	PLI			2.39^**ǂǂ**^	2.55^**ǂǂ**^	1.90^**ǂ**^	2.28^**ǂǂ**^
	SPI			2.20^**ǂǂ**^	2.30^**ǂǂ**^	1.75^**ǂǂ**^	2.08^**ǂǂ**^
	RI			185.42^**ǂ**^	192.65^**ǂ**^	125.78	167.95^**ǂ**^
	TI			3.12^**ǂǂ**^	3.38^**ǂǂ**^	2.56^**ǂ**^	3.02^**ǂǂ**^

*Note:* * = > TEL, ** = > PEL, ^ǂ^ = moderate toxicity, ^ǂǂ^ = high toxicity.

Abbreviations: PEL = probable effect level, PLI = pollution load index, RI = integrated risk index, SPI = weighted sediment pollution index, TEL = threshold effect level, TI = normalised toxicity index.

### 3.2 *Acanthamoeba* Species Identified in the Vashi Creek

**FIGURE 2:**
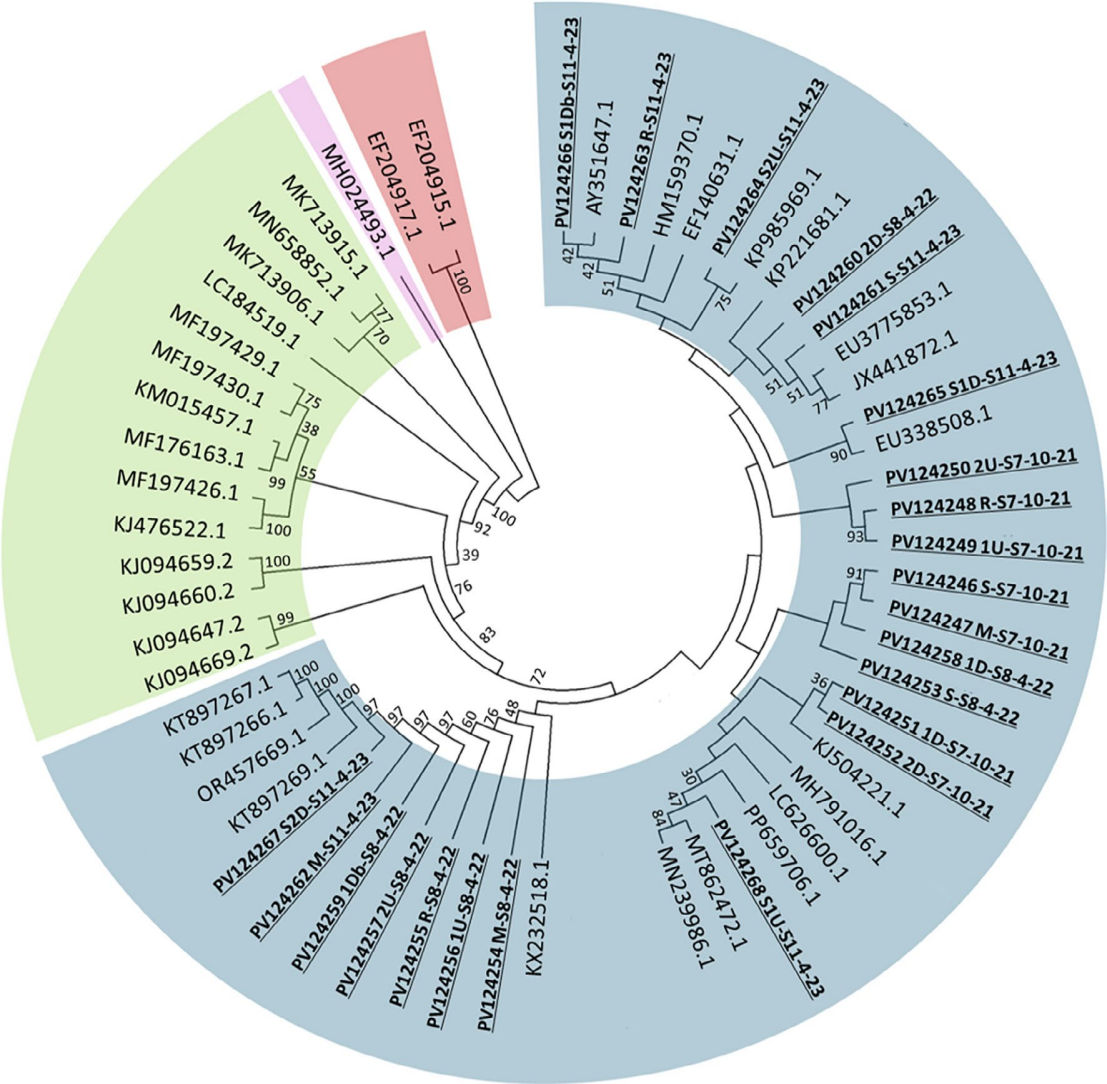
Evolutionary lineage of *Acanthamoeba* isolates from Vashi Creek (highlighted in bold) generated using the ASA.S1 region of the 18S rRNA gene and screened against publicly available sequences (NCBI). All isolates clustered most closely with those from the T4 genotype (highlighted blue), while other genotypes (highlighted green) were not represented in our isolates. Lesser related amoebae were highlighted in pink (Protoacanthamoeba) and red (Entamoeba) and similar regions of the 18S gene used for comparison.

A total of 23 isolates of *Acanthamoeba* were isolated from the Vashi Creek over 3 time points. Interestingly, the isolates all belonged to the T4 genotype, perhaps unsurprising given their documented adaptability when faced with harsh conditions (Ling et al. 2024; Lakhundi et al. 2014; Alves et al. 2017; Siddiqui and Khan 2012). Figure 2 documents the clustering of Vashi Creek isolated amoebae with other T4 isolates, forming 3 distinct clades within the genotype.

### 3.3 Intracellular Bacterial Communities From *Acanthamoeba* Are More Resistant to Antibiotics Than Those From the Surrounding Sediment

**FIGURE 3:**
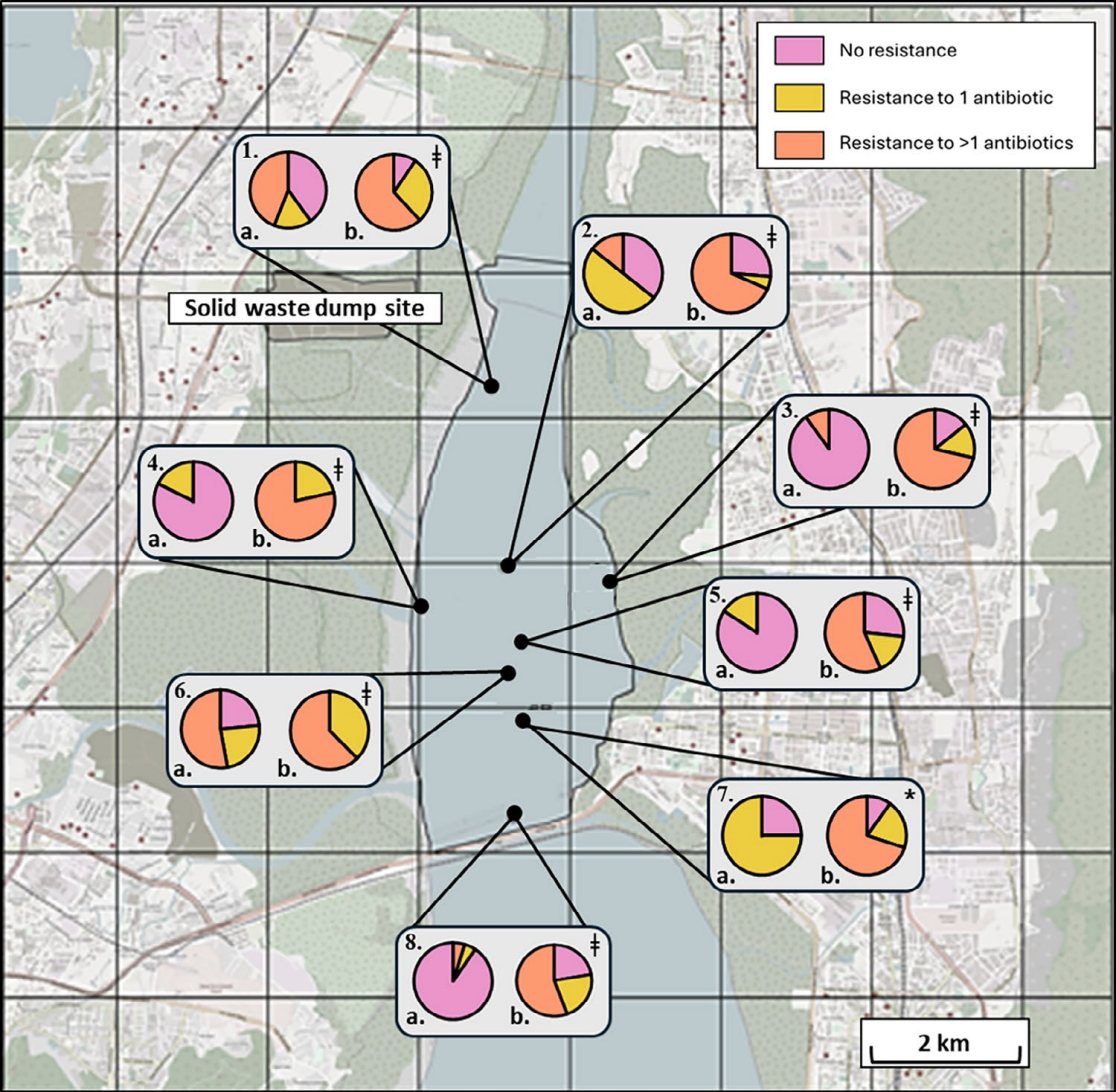
Antibiotic susceptibility of sediment‐associated isolates (a) versus Acanthamoeba‐associated isolates (b) across all sites (1–8). Bacteria showing resistance to none of the antibiotics tested are shown in pink, those resistant to only 1 antibiotic are shown in yellow, and those resistant to multiple antibiotics are shown in orange. *p* < 0.05 denoted by ‘*‘, *p* < 0.0001 denoted by ‘**ǂ**’.

A total of 291 bacterial isolates were selected from either the sediment (134) or amoebae (157) and screened against appropriate antibiotics. Antibiotics were selected based on Gram staining and the agar used for selecting isolates (LB vs. Cetrimide). Figure 3 shows a site‐by‐site overview of the percentage of all isolates showing no resistance, resistance to 1 antibiotic, or resistance to more than 1 antibiotic. Total resistance observed was significantly higher in *Acanthamoeba*‐associated bacterial (AAB) communities at all sites (*p* < 0.0001 for all sites other than Site 7; *p* < 0.05). Multidrug resistant (MDR) isolates (> 1 antibiotic) were also significantly more common in AAB communities, with a 50% or more increase in MDR isolates documented in six of the eight sites relative to sediment‐associated bacterial (SAB) communities (Figure 3; Site 2: 68%:14%, Site 3: 71%:10%, Site 4: 79%:0%, Site 5: 57%:0%, Site 7: 70%:0%). Interestingly, the most pronounced differences in overall resistance were identified close to the shoreline at either side of the creek (Figure 3; Sites 3 and 4).

**FIGURE 4:**
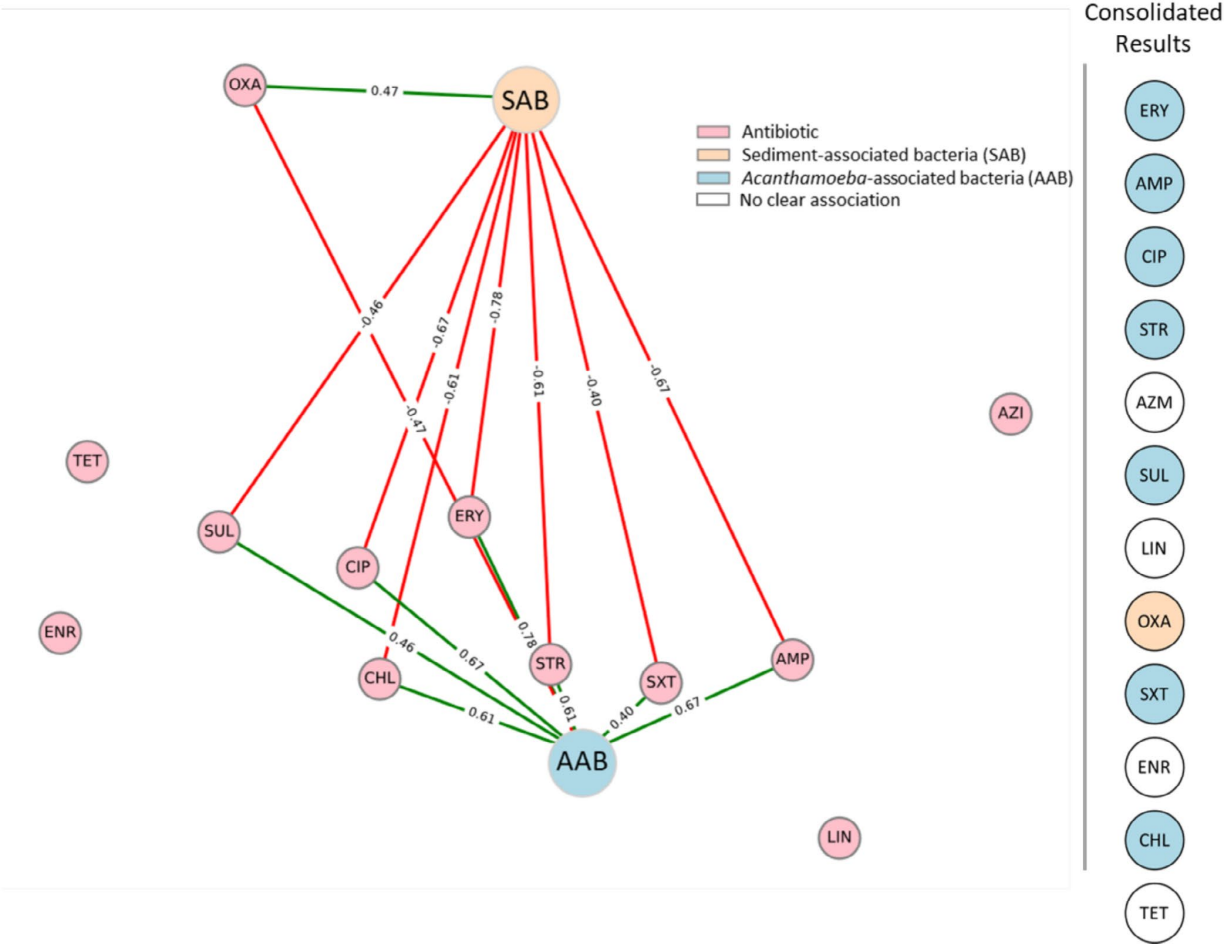
The influence of sediment‐ or *Acanthamoeba*‐association on resistance. Pink nodes represent antibiotics; ampicillin (AMP), azithromycin (AZM), chloramphenicol (CHL), ciprofloxacin (CIP), enrofloxacin (ENR), erythromycin (ERY), linezolid (LIN), oxacillin (OXA), streptomycin (STR), sulfamethazine (SUL), sulfamethoxazole/trimethoprim (SXT), tetracycline (TET). Orange node represents sediment‐associated bacteria (SAB), blue node represents Acanthamoeba‐associated bacteria (AAB), edges denote significant correlations (*r* > 0.4), edge colour represents the type of correlation (Green = positive, Red = negative), edge values represent the correlation coefficient (*r*). Influence of bacterial association on resistance for all antibiotics is summarised (right of grey dividing line; SAB = orange circle, AAB = blue circle, No clear influence = white circle).

*Acanthamoeba*‐associated bacterial communities were more strongly correlated with antibiotic resistance than SAB (Figure 4, *r* > 0.4). Resistance to ampicillin, sulfamethazine, streptomycin, erythromycin, chloramphenicol, sulfamethoxazole/trimethoprim, and ciprofloxacin correlated with AAB, while only oxacillin showed a stronger correlation to bacteria isolated from the sediment. While not meeting the threshold of significance, tetracycline resistance did show a higher correlation with the SAB (*r* = 0.36) and azithromycin to AAB (*r* = 0.37). Linezolid and enrofloxacin showed no significant correlations (*r* = 0.11 and 0.15 respectively).

### 3.4 Antibiotic Susceptibility of Bacterial Isolates

**FIGURE 5:**
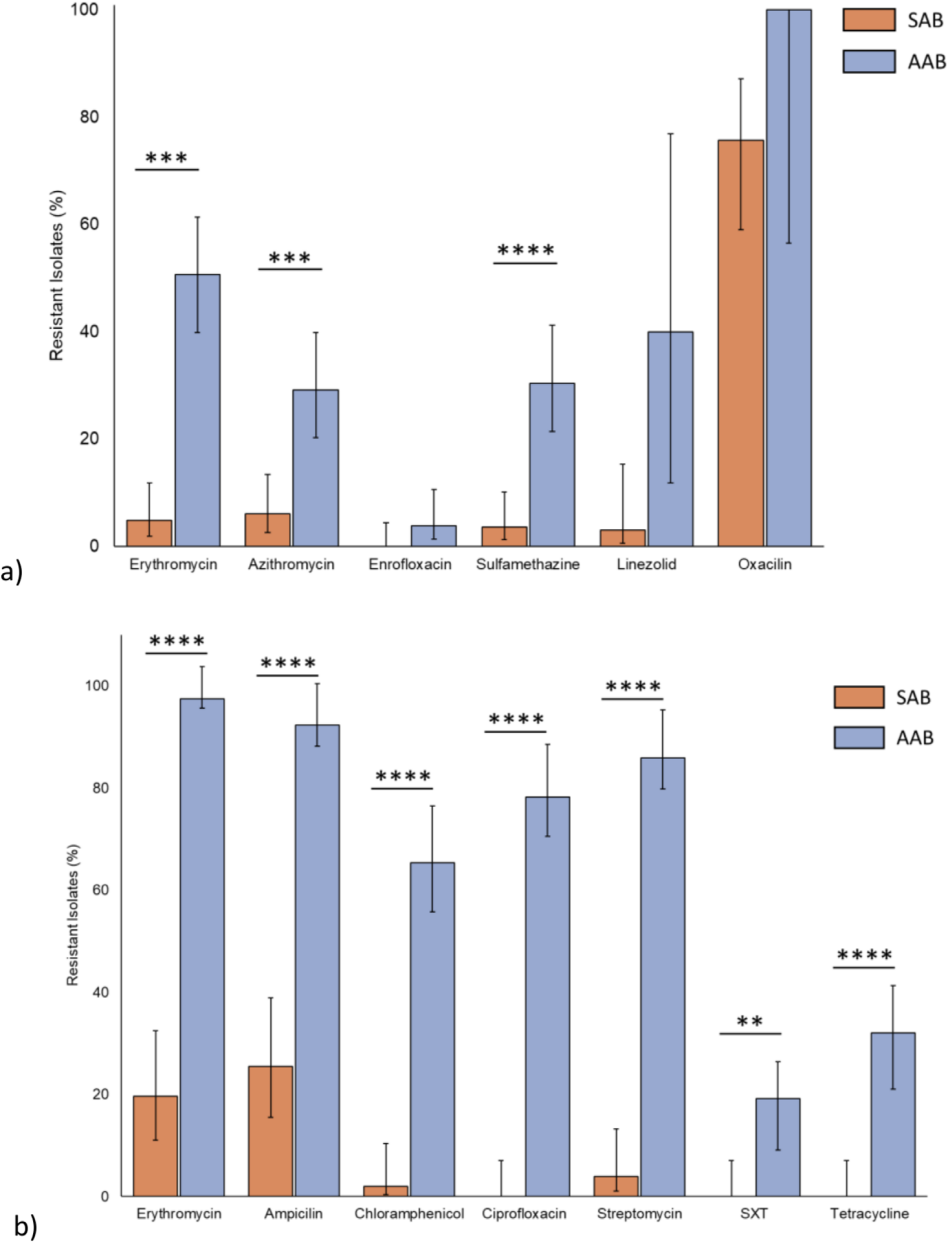
Percent of sediment‐ (orange bars) and *Acanthamoeba*‐associated bacteria (blue bars) resistant to screened antibiotics selected on (a) LB agar; *n* = 162; 83 SAB, 79 AAB and (b) cetrimide agar; *n* = 129; 51 SAB, 78 AAB. Isolates were screened in triplicate. Data are percent of isolates resistant from respective conditions SAB or AAB. Error bars denote 95% confidence interval (Wilson method), significance denoted by ‘**’ for *p* < 0.01, ‘***’ for *p* < 0.001 and ‘****’ for *p* < 0.0001 as determined by Fisher's exact test.

Bacteria from sediment were first selected for using a nutrient‐rich agar (LB agar) and screened in triplicate using six antibiotics: erythromycin, azithromycin, enrofloxacin, sulfamethazine, linezolid, and oxacillin (Figure 5a). Generally, antibiotic resistance was higher in isolates taken from *Acanthamoeba*. Higher levels of resistant isolates were noted in erythromycin (*p* < 0.001), azithromycin (*p* < 0.001) and sulfamethazine (*p* < 0.0001). We then chose to incorporate a selective media (cetrimide) due to the documented relationship of *Acanthamoeba* with Pseudomonads. Bacteria were selected for as before on cetrimide agar, and screened against eight antibiotics (Figure 5b): erythromycin, ampicillin, chloramphenicol, ciprofloxacin, streptomycin, sulfamethoxazole/trimethoprim (SXT), and tetracycline. Interestingly, resistance to all antibiotics was significantly higher in AAB relative to those isolated from the total sediment (*p* < 0.01).

### 3.5 Multiple Antibiotic Resistance Profiles

**FIGURE 6:**
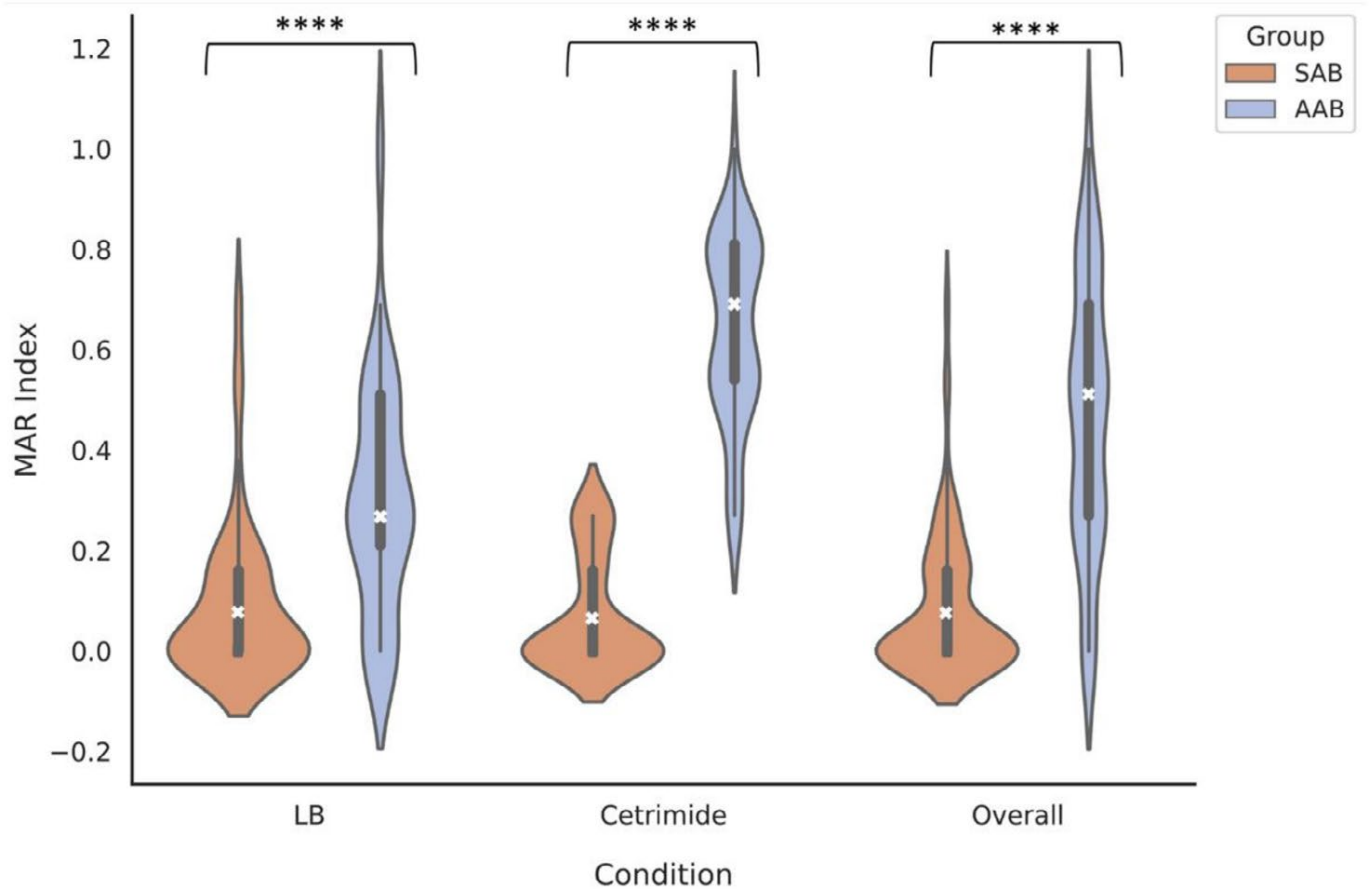
Average multiple antibiotic (MAR) index for isolates selected for on LB agar, cetrimide agar, and combined data from both LB and cetrimide agar conditions. This plot illustrates the distribution of bacterial MAR index in sediment‐associated bacterial communities (SAB; orange) and Acanthamoeba‐associated bacterial communities (AAB; blue). The shaded shapes illustrate the spread and relative frequency of values, where greater width indicates a higher density of isolates with that MAR index. The median is denoted by a white cross, the shaded box shows the interquartile range. Individual points are overlaid to display the underlying data. Sample sizes: SAB (*n* = 83, 51, 134), AAB (*n* = 79, 78, and 157). Significance denoted by ‘****’ for *p* < 0.0001 as determined by the Mann–Whitney *U* test.

The MAR index for all conditions was calculated to compare multidrug resistance capabilities of each isolate. Figure 6 shows that the average MAR index for all conditions was significantly higher in *Acanthamoeba*‐associated communities (LB; 0.31, cetrimide; 0.66, and combined; 0.45, *p* < 0.001) relative to those from the sediment (LB; 0.09, cetrimide; 0.08, and combined; 0.09), with wider distributions of data.

### 3.6 Influence of PTEs on Resistance

**FIGURE 7:**
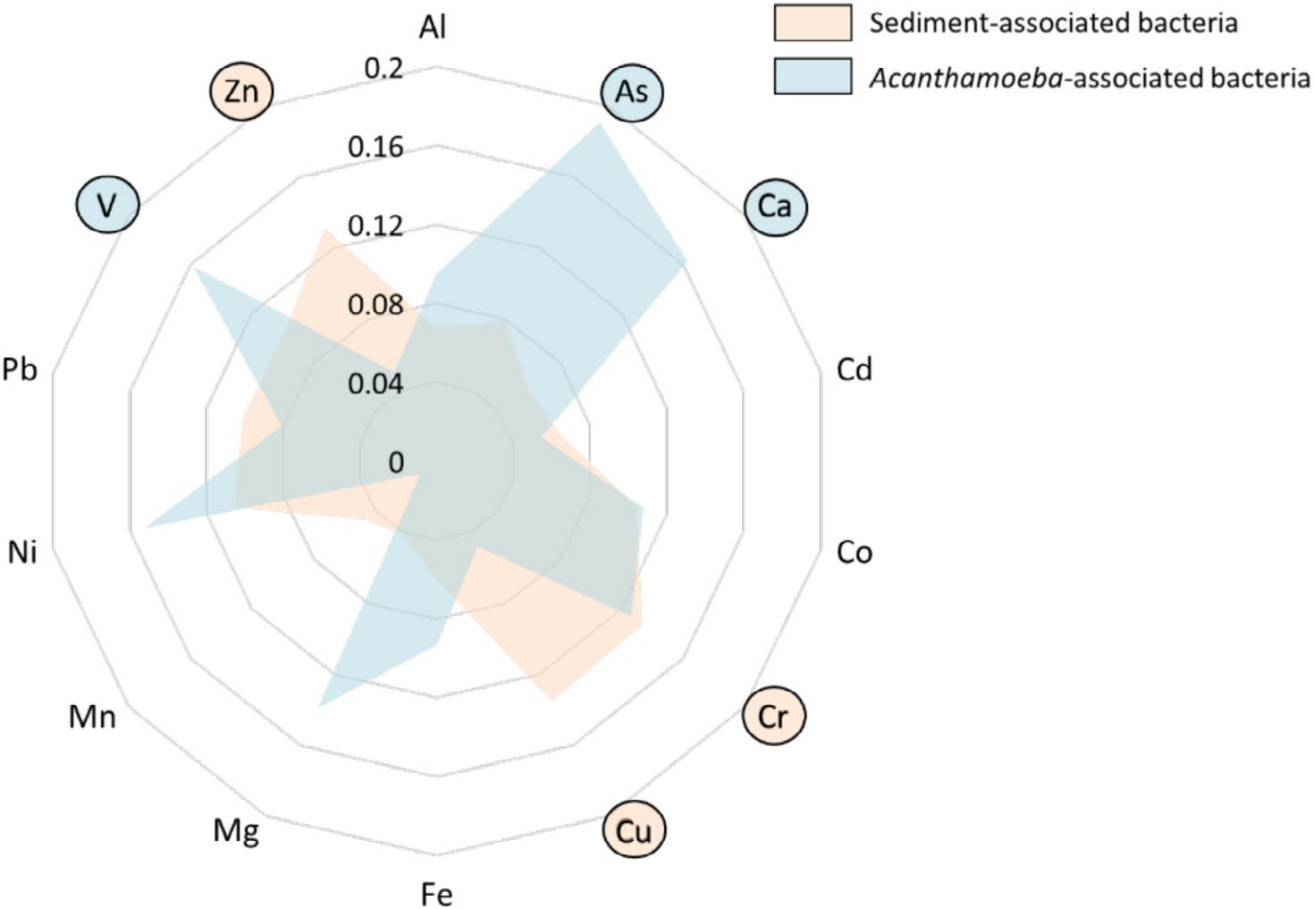
Radar plot summarising correlations of potentially toxic elements (PTEs) with antibiotic resistance in sediment‐associated (SAB; orange) and Acanthamoeba‐associated bacterial communities (AAB; blue). Correlations were weighted and stabilised using Fisher's z transformation, and values were back‐transformed to r for interpretation. Axes represent individual PTEs, with the plotted lines showing the overall strength of association for SAB and AAB. PTEs: Aluminium (Al), arsenic (As), calcium (Ca), cadmium (Cd), cobalt (Co), chromium (Cr), copper (Cu), iron (Fe), magnesium (Mg), manganese (Mn), nickel (Ni), lead (Pb), vanadium (V) and zinc (Zn).

It has been established that the selection of antibiotic resistance traits in the environment can be influenced by the presence of potentially toxic elements. While this is well documented, the potential for predatory protists such as *Acanthamoeba* to harbour a unique resistome has only been postulated to this point, and the driving factors that could contribute to that variability within the cell have yet to be considered. Using the resistance and PTE data generated previously, we sought to identify potential correlations that could provide more insight into the relationship between PTEs and resistance (Figure 7 and Figure S1). In SAB isolates, zinc, chromium, and copper had the biggest impact on resistance (Figure 7). Of particular interest is the strong positive correlation between zinc and chloramphenicol resistance (*r* = 0.759). Chloramphenicol resistance was also positively associated with cadmium (*r* = 0.5) and chromium (*r* = 0.482) in these isolates (Figure S1), whereas in AAB isolates, chloramphenicol was among the least influenced by the PTEs in the environment, only showing a weak association with arsenic (Figure S1; *r* = 0.345). Unlike the sediment‐associated isolates, copper and zinc appear to have almost no influence on resistance traits observed within the amoeba‐associated isolates (Figure 7), and chromium is primarily negatively associated with resistance (Figure S1). Instead, the most significant influencers appear to be arsenic, vanadium, and calcium. Arsenic in particular is noteworthy, having positive associations with 5 of the antibiotics screened: ampicillin (*r* = 0.453), ciprofloxacin (*r* = 0.4), sulfamethoxazole/trimethoprim (*r* = 0.414), chloramphenicol (*r* = 0.345), and tetracycline (*r* = 0.339), while having no positive influence on the SAB resistance profile (Figure S1).

## 4 Discussion

Anthropogenic pollutants significantly impact microbial communities, with PTEs selecting for antibiotic resistance through cross‐resistance mechanisms and co‐localisation of resistance genes on mobile genetic elements (Baker‐Austin et al. 2006). However, the role of predatory protists, such as *Acanthamoeba*, in influencing bacterial resistance has received less attention. These amoebae are resilient organisms that harbour unique intracellular microbiomes, which can differ from their surrounding environments (Mooney et al. 2024, 2025; Sriram et al. 2008; Chambers and Thompson 1976). Beyond this, the role of potentially toxic elements in the distribution and diversity of *Acanthamoeba* has not been fully explored, nor has the role of these compounds in the selection of antibiotic resistant endosymbionts, an issue that underscores the necessity of a One Health perspective in addressing AMR within interconnected environmental and public health systems.

Vashi Creek, a tidal estuarine system in Navi Mumbai, India, is subject to substantial anthropogenic pollution (Corbett et al. 2025). Our screening of eight sites across three sampling events (SE1: Nov 2021, SE2: Apr 2022, SE3: Apr 2023) revealed consistent exposure to PTEs at levels exceeding recommended guidelines (Canadian Council of Ministers of the Environment 2002; Macdonald et al. 1996). Arsenic, cadmium, copper, and zinc frequently surpassed their threshold effect levels (TEL), while chromium and nickel exceeded probable effect levels (PEL). Toxicity indicators classified the system as highly toxic during SE1 and SE2 and moderately toxic during SE3, suggesting persistent environmental stress on microbial communities. Despite this toxic environment, viable *Acanthamoeba* were present at all sites. These amoebae, capable of forming structurally resilient cysts, are well adapted to extreme conditions and have high tolerance for many commonly used antimicrobials (Mooney et al. 2024; Johnston et al. 2009; Coulon et al. 2010; Lakhundi et al. 2014; Lemgruber et al. 2010; Hasan et al. 2021). Currently, *Acanthamoeba* are categorised into 23 distinct genotypes (T1–T23) based on differences in their 18S rRNA gene sequences (Schroeder et al. 2001; Rayamajhee et al. 2022). Many genotypes are not recognised pathogens, yet some are capable of causing severe human infections (Lorenzo‐Morales et al. 2015; Siddiqui and Khan 2012; Jones et al. 1975). Of the clinically relevant *Acanthamoeba* genotypes, those belonging to the T4 group are most commonly reported (Maciver et al. 2013). Our findings revealed that all 23 isolates belonged to the T4 genotype, known for their resilience under harsh chemical and physical stressors (Mooney et al. 2024, 2025; Lakhundi et al. 2014; Alves et al. 2017) and this may explain the observed dominance within this highly polluted system. From a public health perspective, this is concerning: T4 is the genotype most commonly associated with keratitis and granulomatous amoebic encephalitis (Maciver et al. 2013; Diehl et al. 2021), and the potential for T4 amoebae to act as reservoirs and vectors of multidrug‐resistant bacteria increases the risk of zoonotic or environmental spillover. These findings reinforce the need to integrate protist surveillance into One Health AMR frameworks, as polluted ecosystems may not only enrich bacterial resistomes but also promote the persistence of clinically significant amoebae genotypes.

Additionally, *Acanthamoeba* play a crucial ecological role by predating bacteria, contributing to microbial diversity. However, this predator–prey relationship has led to the evolution of phagocyte‐resistant bacteria capable of intracellular survival (Rayamajhee et al. 2024, 2021; Sarink et al. 2020; Dobrowsky et al. 2017). Such bacteria, termed *Acanthamoeba*‐associated bacteria (AAB), may be less detectable and could potentially be more resistant to antibiotics than sediment‐associated bacteria (SAB). Our study examined culturable bacteria using a broad‐spectrum nutrient‐rich agar (LB) and a selective agar (Cetrimide) due to the documented association between *Acanthamoeba* and Pseudomonads (Mooney et al. 2024, 2025; Dey et al. 2019; José Maschio et al. 2015). The methodological approach used here, based on culturable isolation and phenotypic antibiotic susceptibility, deliberately avoids taxonomic profiling to maximize relevance in LMIC contexts. These regions face the dual burden of high AMR risk and limited infrastructure for advanced microbial diagnostics. Our workflow, by contrast, is cost‐effective, requires minimal technical infrastructure, and can be adapted for routine environmental monitoring. The antibiotic susceptibility testing revealed significant differences between AAB and SAB resistance profiles. AAB exhibited higher resistance to streptomycin, ampicillin, ciprofloxacin, chloramphenicol, sulfamethazine, erythromycin and sulfamethoxazole/trimethoprim, while SAB showed a stronger association with oxacillin resistance. A limitation of this study is its reliance on culture‐dependent profiling, which may bias isolate recovery towards readily culturable taxa and precludes direct characterization of the broader microbial community or resistome. While this approach was deliberately chosen for its scalability and accessibility in low‐resource contexts, it inevitably constrains taxonomic resolution and mechanistic interpretation of resistance. Future work will complement these phenotypic assays with 16S rRNA sequencing and metagenomic resistome analysis to confirm species identity, explore functional resistance determinants, and evaluate the extent to which our culture‐based profiles reflect the total resistome present in amoeba‐associated and sediment‐associated communities.

While the mechanisms underlying this increased resistance remain unclear, they could be the result of metabolic adaptations occurring to permit intracellular survival (Thomas et al. 2010); for example, altered metabolism may limit the efficacy of antibiotics targeting metabolic pathways, such as ciprofloxacin, which inhibits DNA gyrase and topoisomerase IV (Shariati et al. 2022; Campoli‐Richards et al. 1988). Furthermore, intracellular survival may upregulate efflux pumps and antioxidants that contribute to resistance, which may be maintained transiently outside *Acanthamoeba* (Mehta et al. 2016; Cavinato et al. 2020; Whittle et al. 2024; Allgood et al. 2023), while some bacteria within *Acanthamoeba* enter a biofilm‐like state (Lopez et al. 2023; Garcia‐Medina et al. 2005; Kember et al. 2022), potentially conferring protection against antibiotics. This has been observed in *Pseudomonas aeruginosa* incubated within bladder epithelial cells (Penaranda et al. 2021). The intracellular environment can also promote genetic adaptations through increased mutation rates (Hasan et al. 2021) and may influence horizontal gene transfer, though further research is needed to confirm this.

AAB also exhibited significantly higher multidrug resistance. The multiple antibiotic resistance (MAR) index, which quantifies resistance across multiple antibiotics (Krumperman 1983), was 5.6 times higher in AAB (0.45) than in SAB (0.08). Notably, 46% of AAB isolates were resistant to four or more antibiotics, compared to only 0.6% of SAB isolates. Additionally, 22% of AAB isolates were resistant to six or more antibiotics. This raises concerns given that *Acanthamoeba* are known to shield potential pathogens from detection in high‐risk environments (Mooney et al. 2024), and that many *Acanthamoeba* infections are often accompanied by bacterial co‐infections (Rayamajhee et al. 2024, 2021; Okubo et al. 2018; Fukumoto et al. 2016). These findings support the ‘training ground’ hypothesis, which suggests that survival within amoebae selects for bacteria with increased clinical significance due to enhanced virulence or reduced antibiotic susceptibility (Henriquez et al. 2021). The protective role of *Acanthamoeba* for intracellular bacteria has been previously demonstrated, but its contribution to resistance selection remains uncertain. Recent work found that *P. putida* exposed to ciprofloxacin in the presence of *Acanthamoeba* resulted in cells 40‐fold more resistant to ciprofloxacin and with increased resistance to several other antibiotics (Giammarini et al. 2025), similar to findings in mammalian cells (Penaranda et al. 2021). This could indicate that sub‐lethal exposure within amoebae might select for AMR in the environment, and aligns with the findings presented here.

The higher MAR indices in *Acanthamoeba*‐associated isolates may reflect both enrichment of resistant taxa and intracellular modulation of resistance. An important methodological consideration is that cetrimide agar enriches for *Pseudomonas* spp., which are intrinsically more resistant to many antibiotics, potentially inflating absolute resistance rates. However, the consistent pattern of increased resistance in AAB across both LB and cetrimide cohorts, including significantly higher resistance to all screened antibiotics in the *Pseudomonas*‐enriched subset, indicates that medium‐related or compositional bias alone cannot account for the effect. These findings suggest that intracellular residence within *Acanthamoeba* contributes additional resistance phenotypes beyond those expected from community composition. However, it is important to emphasize that our comparisons are community‐level and do not control for taxonomic composition. Therefore, while AAB displayed higher resistance and MAR indices than SAB, we cannot conclude that identical bacterial species are more resistant within amoebae.

The structure and function of microbial communities are shaped by complex interactions between environmental stressors and microbial composition (Orland et al. 2019). Given the influence of environmental factors on resistance, we examined whether PTEs in Vashi Creek affected antibiotic resistance in AAB and SAB comparatively. Among the screened PTEs, vanadium, arsenic, chromium and nickel were the strongest contributors to resistance. Arsenic, chromium and nickel have been linked to antibiotic resistance via efflux pump upregulation, particularly in tetracyclines, fluoroquinolones, aminoglycosides, macrolides, amphenicols and β‐lactams (Li et al. 2015; Nguyen et al. 2023; Haque et al. 2024; Gillieatt and Coleman 2024). Vanadium, though less frequently studied, also induces efflux pump expression and alters membrane permeability, potentially contributing to resistance (Aureliano et al. 2023; Aendekerk et al. 2002). Interestingly, the influential PTEs varied between AAB and SAB. Zinc, copper and chromium showed the strongest correlation with SAB resistance, particularly chloramphenicol resistance. While this relationship is not well documented in environmental samples, in vitro studies have demonstrated co‐resistance between zinc and chloramphenicol (Rihacek et al. 2023). In contrast, AAB chloramphenicol resistance was weakly associated with arsenic but no other PTEs, whereas ampicillin resistance correlated with seven PTEs (Al, As, Cr, Mg, Ni, Pb and V). This suggests enhanced efflux activity (Baker‐Austin et al. 2006; Levy 2002; Nies 2003), upregulated stress response pathways (Kaviani Rad et al. 2022; Ahmad 2022), or co‐selection of resistant genes (Kariuki et al. 2015; Wu et al. 2018).

Unlike SAB, the biggest drivers influencing increased antibiotic resistance in AAB were arsenic, vanadium and calcium. Interestingly, these PTEs can have a significant influence on intracellular survival within phagocytic cells. For example, amoebae have been shown to use arsenic to aid in the breakdown of ingested bacteria, and that resistance to arsenic can increase survivability within cells (Hao et al. 2017). Arsenic resistance has been closely associated with resistance to ciprofloxacin, tetracycline, chloramphenicol and ß‐lactams via shared resistance mechanisms (Baker‐Austin et al. 2006; Nies 2003; Wright 2005; Silver and Phung 1996; Mukhopadhyay and Rosen 2002), and it is interesting to note that our observations are in agreement with this in AAB. Additionally, we also noted a significant relationship between arsenic and resistance to trimethoprim/sulfamethoxazole, which could be the result of reduced membrane permeability or enhanced efflux pump activity (Eliopoulos and Huovinen 2001). The role of calcium in the resistance of AAB but not SAB is of particular interest. Calcium is a critical secondary messenger in host cells, regulating phagocyte responses (Pradhan et al. 2019); however, these signalling pathways are often manipulated by intracellular bacteria to resist digestion by the cell (Andersson et al. 1999; Li et al. 2021) Additionally, calcium has also been shown to promote biofilm formation (Tischler et al. 2018), perhaps permitting intracellular survival and limiting drug efficacy. The association of calcium with intracellular resistance suggests an enhanced selection for bacteria capable of manipulating host calcium‐dependent pathways, and that this might also confer resistance through metabolic adaptations within *Acanthamoeba*. Vanadium has also been shown to increase the production of reactive oxygen species (ROS) (Aureliano et al. 2023), and it could be argued that, given the importance of ROS in the breakdown of ingested bacteria, resistance to vanadium confers some increased resistance to phagosome activity and antibiotics, be this through changes in membrane permeability or in the upregulation of antioxidants or key proteins (Aureliano et al. 2023; Aendekerk et al. 2002). Our initial observations suggest that PTEs important for intracellular survival, particularly with regards to the phagocytic ability of the amoeba, are the most significant drivers of antibiotic resistance in intracellular bacteria in the environment. However, these correlations do not establish causality; stronger associations in AAB may reflect intracellular selection or co‐occurrence with elevated metal levels. Experimental validation through controlled exposure assays will be required to determine whether these elements directly induce resistance. Another ecological consideration is that, as motile organisms, amoebae can acquire intracellular bacteria from other systems, meaning that the communities recovered may not directly represent the sediment microbiota at the sample site (Mooney et al. 2024, 2025). This mobility also raises the possibility that amoebae might act as vectors, transporting resistant bacteria across spatial and ecological boundaries. Future studies employing genomic profiling and spatially resolved sampling will be necessary to determine the extent to which amoebae redistribute multidrug‐resistant bacteria within polluted ecosystems.

Despite high environmental toxicity, *Acanthamoeba* persist and harbor antibiotic‐resistant bacteria. The dominance of the clinically relevant T4 genotype in polluted environments is concerning. More alarming, however, is the role of these amoebae as reservoirs for multidrug‐resistant bacteria. The resistome of AAB appears uniquely influenced by PTEs affecting phagocytosis, rather than those influencing SAB. These findings not only advance our understanding of intracellular AMR evolution under pollution stress but also have direct implications for AMR surveillance and could be integrated into existing One Health monitoring frameworks. Current global initiatives such as the World Health Organization (WHO) Global Antimicrobial Resistance Surveillance System (GLASS) (World Health Organization (WHO) 2022) and the WHO Tricycle Protocol (World Health Organization (WHO) 2021) focus mainly on clinical and agricultural isolates, with limited attention to environmental reservoirs. The Joint External Evaluation (JEE) tool under the International Health Regulations (IHR) (World Health Organization 2022) and the United Nations Environment Programme (UNEP) (United Nations Environment Programme (UNEP) 2023) report on environmental dimensions of AMR also highlight the need for environmental indicators, yet lack clearly defined biological sentinels. We propose that amoeba‐associated bacteria serve as sentinel indicators of multidrug resistance, complementing genomic surveillance with a low‐cost, phenotypic approach. At the regional and national levels, frameworks such as the European Antimicrobial Resistance Surveillance Network (EARS‐Net) (European Centre for Disease Prevention and Control (ECDC) and ‘European Antimicrobial Resistance Surveillance Network (EARS‐Net).’ 2010) and India's National Action Plan on AMR (Government of India M of H and FW 2017) already acknowledge environmental contributions, providing clear opportunities for integration. Embedding protist–bacteria interactions into these systems would enable earlier detection of environmentally derived resistance threats, particularly in low‐ and middle‐income countries where surveillance capacity is limited.

Building on the potential integration of protist monitoring into global AMR surveillance frameworks, several avenues for future research should be pursued. Future studies should incorporate 16S rRNA sequencing and resistome analysis to confirm bacterial taxa and determine whether elevated resistance persists within matched species. Controlled metal‐exposure assays are also needed to establish whether the observed correlations reflect true selective pressures or environmental co‐occurrence. Finally, integration into One Health surveillance frameworks should be pursued to enhance interpretability and policy relevance. These steps will be important to define the mechanisms underlying amoebae‐associated resistance and to gauge its wider implications for environmental and public health.

## Author Contributions

Conceptualization: F.L.H, S.M., S.M., A.H., J.C., R.M. Methodology: R.M., F.L.H., K.R. Investigation: R.M., E.C., E.G., K.R., C.D., E.M., A.T.A.A., A.R., P.S.V., H.K.W., J.S. Resources: S.M., S.M., A.T.A.A., A.R., P.S.V., H.K.W., J.S. Data curation: R.M., E.C., K.R. Writing: original draft: R.M., F.L.H. Writing: review and editing: F.L.H., S.M., S.M., A.H., J.C., R.M., K.R. Visualization: R.M., E.C., K.R. Supervision: F.L.H., S.M., S.M., A.H., J.C. Project administration: F.L.H., S.M., S.M., A.H., J.C., R.M. Funding acquisition: F.L.H., S.M., S.M., A.H., J.C.

## Conflicts of Interest

The authors declare no conflicts of interest.

## Supporting information


**Figure S1:** Correlations of PTEs with antibiotic resistance in sediment‐associated bacteria and Acanthamoeba‐associated bacteria. Colour intensity of the cells represent the strength and direction of the correlation: positive correlations are shown in red, and negative correlations are shown in blue, non‐significant correlations are shown in white. Correlations were assessed across aggregated sampling events. Variable pairs that met both exploratory statistical thresholds (*p* < 0.1) and practical significance thresholds (*r* > 0.3) are highlighted as follows; *r* > 0.3 (*), *r* > 0.4 (**), *r* > 0.6 (***). The scale bar on the right indicates the magnitude of the correlation. Antibiotics; ampicillin (AMP), and tetracycline (TET), azithromycin (AZM), chloramphenicol (CHL), ciprofloxacin (CIP), enrofloxacin (ENR), erythromycin (ERY), linezolid (LIN), oxacillin (OXA), streptomycin (STR), sulfamethazine (SUL), sulfamethoxazole/trimethoprim (SXT).

## Data Availability

The data that supports the findings of this study are available in the Supporting Information of this article.
