# Supplementary figures and images for: The Microbial Trojan Horse and Antimicrobial Resistance: Acanthamoeba as an Environmental Reservoir for Multidrug Resistant Bacteria
Source: Environ Microbiol. 2025 Oct 29;27(11):e70193. doi: 10.1111/1462-2920.70193 (PMC12572456; doi:10.1111/1462-2920.70193)

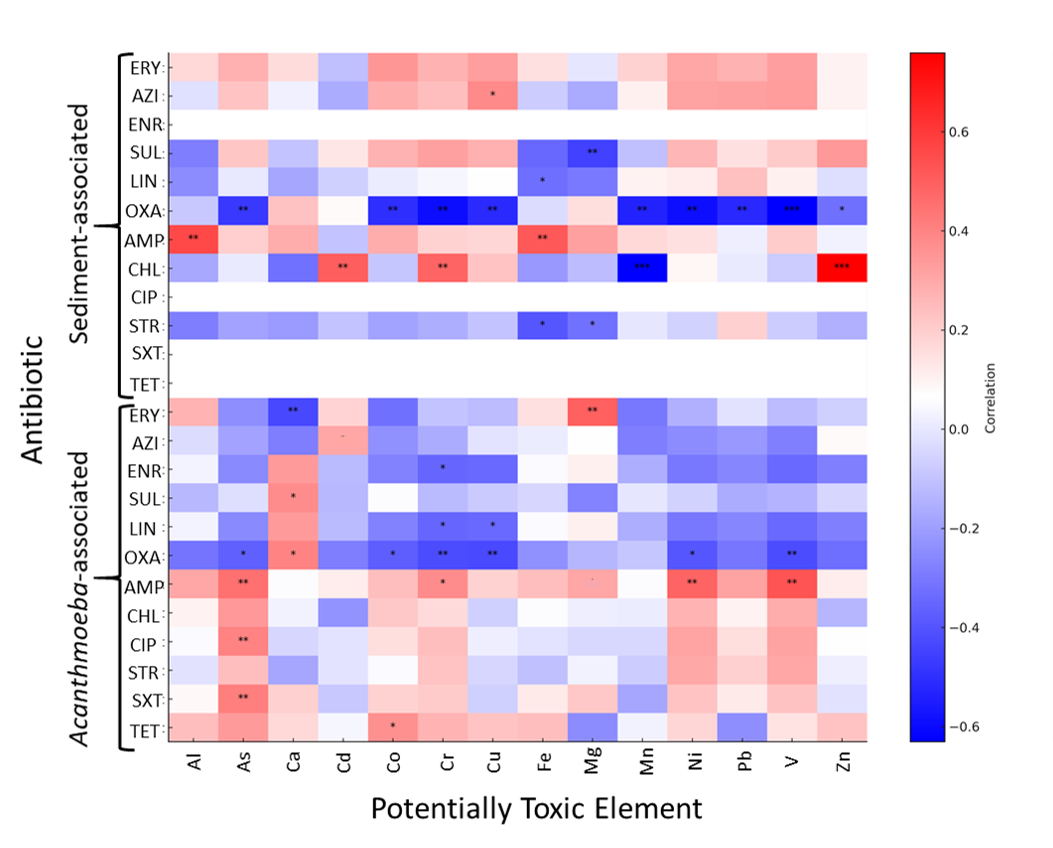

Supplement: Supplementary file 1 — Figure S1: Correlations of PTEs with antibiotic resistance in sediment‐associated bacteria and Acanthamoeba‐associated bacteria. Colour intensity of the cells represent the strength and direction of the correlation: positive correlations are shown in red, and negative correlations are shown in blue, non‐significant correlations are shown in white. Correlations were assessed across aggregated sampling events. Variable pairs that met both exploratory statistical thresholds (p < 0.1) and practical significance thresholds (r > 0.3) are highlighted as follows; r > 0.3 (*), r > 0.4 (**), r > 0.6 (***). The scale bar on the right indicates the magnitude of the correlation. Antibiotics; ampicillin (AMP), and tetracycline (TET), azithromycin (AZM), chloramphenicol (CHL), ciprofloxacin (CIP), enrofloxacin (ENR), erythromycin (ERY), linezolid (LIN), oxacillin (OXA), streptomycin (STR), sulfamethazine (SUL), sulfamethoxazole/trimethoprim (SXT). [file EMI-27-e70193-s001.png]
